# Prevalence and characteristics of registered falls in a Belgian University Psychiatric Hospital

**DOI:** 10.3389/fpubh.2022.1020975

**Published:** 2022-10-28

**Authors:** Lynn de Smet, Arnout Carpels, Lotte Creten, Louise De Pauw, Laura Van Eldere, Franciska Desplenter, Marc De Hert

**Affiliations:** ^1^University Psychiatric Center, KU Leuven, Leuven, Belgium; ^2^Public Psychiatric Care Center Rekem, Rekem, Belgium; ^3^Clinical Pharmacology and Pharmacotherapy, Department of Pharmaceutical and Pharmacological Sciences, KU Leuven, Leuven, Belgium; ^4^Center for Clinical Psychiatry, Department of Biomedical Sciences, KU Leuven, Leuven, Belgium; ^5^Antwerp Health Law and Ethics Chair, Universiteit Antwerpen, Antwerp, Belgium

**Keywords:** falls, fall risk, fall prevention, psychiatric setting, descriptive statistics

## Abstract

**Objectives:**

Falls in elderly patients treated in general hospitals have already been the focus of several studies. Research within psychiatric settings, however, remains limited, despite the fact that this population has a number of characteristics that could increase the fall risk. The aim of this retrospective study was to estimate the prevalence of falling in patients with a psychiatric hospital setting.

**Methods:**

A retrospective descriptive chart review of falls registered in the period July 1, 2013 until June 30, 2019 in a Belgian University Psychiatric Hospital was conducted. Data were collected from the “patient related incident report and management system” (PiMS) of the hospital. All registered falls of all hospitalized patients were included in the study.

**Results:**

During the 6-year study period an incidence of 4.4 falls per 1,000 patient days was found. Only 0.5% of the falls resulted in severe injury and none of these falls were fatal. Eighty percent of falls involved a patient over the age of 65. Only 25.0% of the elderly patients suffered physical consequences, while injuries were present in 31.4% of adults and 68.2% of young patients. The two most common causes of a fall were the health status (63.3%) and the behavior (55.1%) of the patient.

**Conclusion:**

The estimated prevalence of falls in our study was generally in line with the rates found in literature on falls in psychiatric settings. Falls in psychiatric settings occur both in younger and older patients, suggesting that all age categories deserve sufficient attention in fall prevention policies. However, more research is necessary to improve fall prevention policies.

## Introduction

Falling remains an important issue in health care. According to the World Health Organization, 684,000 people each year die because of a fall, making it the most common cause of death by non-incidental injuries other than traffic incidents (1). Worldwide, every second a person over the age of 65 falls and every 19 min a person over the age of 65 dies because of the consequences of a fall (2). In these individuals falls are the leading cause of fatal and non-fatal unintentional injuries and are therefore a major burden on the healthcare network (3).

In-hospital falls are associated with extended length of stay, higher health costs and a higher proportion of transfers to nursing home facilities on the long term (4). Besides financial consequences, in-hospital falls can have a negative psychological (fear of falling, reduced confidence in one's own mobility) and social (isolation) impact (5).

The majority of falls have a multifactorial etiology, with intrinsic as well as extrinsic factors increasing the risk of falling. Intrinsic risk factors include a history of falls, acute or chronic illness, pain, frailty, age and insomnia. Extrinsic risk factors can be environmental (bad lighting, slippery floors, loose wires, untied shoe laces, loose carpets, lack of handrails, …) or medication related. Certain medications, both psychotropic and somatic, have been consistently associated with increased fall risks in population-based studies (6, 7). Medication use can result in side effects that may increase the fall risk, but can also reflect on the patient's health status which in turn can increase the fall risk (8). More and more this risk is not only attributed to polypharmacy [intake of at least 5 different drugs (9)], but mainly to the intake of the so-called Fall Risk Increasing Drugs (FRID: antidepressants, antipsychotics, antihypertensive drugs, narcotic analgesics, antiparkinson medication, hypnotics, benzodiazepines, antidiabetics, antiepileptics) (10, 11). Poorly educated caregivers and the absence of a fall prevention policy are also considered as extrinsic risk factors (12).

A recent study showed that working age adults using mental health services had almost four times the incidence of hospitalized falls compared to a general population (13). Stubbs et al. (14) found that people with schizophrenia have a 50–100% increased risk of fracture compared to people without mental illness. Chu et al. (15) found a significantly higher risk for hip and vertebral fractures in people with schizophrenia compared to controls. Zhu et al. (16) recently showed that elevated depressive symptoms in Chinese people are associated with an increased risk for falls and hip fractures. A recent Swedish study showed that the risk of injurious falls is increased in both women and men with eating disorders (17). The higher incidence of falls in this psychiatric population can partially be explained by the high use of psychotropic medication. These drugs, as mentioned above, can have side effects such as dizziness, orthostatic hypotension, decreased alertness and sedation, which can increase the fall-risk (18, 19). In addition, hospitalized psychiatric patients are generally more mobile than patients in a general hospital and also more likely to be restless, agitated and disoriented, which can also increase the risk of falls (20, 21). Chan et al. identified severe extrapyramidal symptoms, more severe psychotic symptoms, higher doses of benzodiazepines and adjusting medication in the 24 h time interval before the fall as risk factors in an inpatient psychiatric population (without any specific age category). Recurrent falling was associated with symptoms of parkinsonism, psychiatric comorbidities and lower extremity movement restrictions (18). A recent study in Thailand showed that an acute psychotic condition, polypharmacy with more than four types of medicines and taking atypical psychiatric drugs are associated with increased inpatient falls (22).

Regarding the prevalence of inpatient falls, the existing literature focused mainly on falls in elderly (23–29) and patients in general hospitals (5, 11, 30–34). Fall ratio's (expressed as falls per 1,000 patient days) between 1.70 and 3.56 were found, with a lower incidence on surgical than non-surgical wards (30–33, 35). A recent study on fall prevalence in Veterans Health Administration hospitals in the USA showed a fall ratio of 4.80 falls per 1,000 bed days. (36). A recent systematic review and meta-analysis on the prevalence of falls in psychiatric inpatients in China showed a prevalence of 3% of falls in adult and 7.3% of falls in older adult inpatients (37). A study conducted in 2017 in Brazil showed a fall ratio of 3.7 on the psychiatric ward of a university hospital, being twice as high than the ratios of the other wards of this hospital combined (surgical, non-surgical, emergency) (30). The mean age of psychiatric patients with a fall (56.3 years) was lower than the mean age of medical-surgical patients (65–83 years) (20, 38). There's a lack of qualitative research that focuses on falls (and patient safety in general) in psychiatric patients (30, 39–41).

The aim of this retrospective descriptive study was to map the prevalence of falls in our hospital and to perform a descriptive analysis of the registered falls in order to identify factors that were frequently associated with falling, repeated falling and falling resulting in injury.

## Materials and methods

### Study design and setting

A retrospective descriptive chart review of falls, registered in the period July 1, 2013 up to and including June 30, 2019 in the University Psychiatric Hospital KU Leuven (UPC KU Leuven) (Belgium), was conducted.

The UPC KU Leuven is a Belgian University Psychiatric Hospital consisting of two hospital sites, Kortenberg (KB) and Leuven (LV), both with respectively 446 and 115 beds. Both campuses are located in the same province in Belgium (Vlaams-Brabant).

Approval by the Ethical Research Committee of UZ/KU Leuven was obtained (reference MP011646).

### Collection of data

In the hospital a “patient related incident report and management system” (PiMS) is used to register multiple types of incidents such as falls, medication incidents or aggression (42). Per type of incident a specific electronic registration form is available to document the circumstances and consequences of the incident. Caregivers need to complete the registration form as soon as possible after they encounter an incident. Consequently, these PiMS reports give a complete overview of information regarding patient-related incidents, including falls.

Data of the PiMS reports on falls were provided anonymously by the hospital's quality coordinator. All PiMS reports on fall incidents of all hospitalized patients (both full-time and day admissions) completed during the study period were included. PiMS reports on near incidents were excluded from this analysis.

Data on the number of patient days and the number of admissions were obtained using the hospital's medical registration system. This information was provided anonymously by the hospital's management information report service.

### Outcome

The primary outcome was the prevalence of falls, expressed as falls per number of admissions and falls per 1,000 patient days.

The secondary outcome was the prevalence of falls resulting in injury. Physical injuries were defined as none (incident without injury), mild (intervention required to rule out injury), moderate (incident resulting in temporary injury requiring intervention and/or prolonged hospitalization), severe (incident resulting in permanent injury and, if necessary, requiring intervention to manage a life-threatening situation), and fatal (incident resulting in patient death).

The third outcome was the investigation of the factors (cause and location of the fall, sex and age of the patient) associated with falls and falls resulting in injury. Data on cause and location of the fall were available in the datasets of both campuses, data on age and sex of the patient were only available in the dataset of campus Kortenberg. Age groups were defined as follows: young people (<18 years), adults (18–65 years), youngest elderly (65–74 years), middle elderly (75–84 years), and oldest elderly (>85 years).

The fourth outcome was the prevalence of repeated falls and the factors associated with it. It was also examined how the chance of recurrence was estimated, by looking at the answers in de PiMS form of the previous incident. As mentioned above we could only perform these analyses on the subsample of campus Kortenberg.

### Data analysis

PiMS on registered falls were extracted anonymously and exported to a Microsoft Excel (Seattle, WA, VS) by the hospital's quality coordinator. The two datasets, one for each campus, were merged (Supplementary Figure 1 shows a detailed overview of data handling and processing).

Coding and statistical analysis were done using Microsoft Excel 2010 and SSPS statistical analysis software, respectively. Descriptive statistics were performed to calculate frequencies: percentages and ratios for nominal variables, means and standard deviations for continuous variables.

The prevalence of falls was determined on the basis of the information obtained about the number of falls and the number of patients with a fall and the number of hospitalization days and admissions. For each patient the time between 2 registered falls was calculated. If this period did not exceed 12 months falls were considered as repeated falls.

Due to a more anonymized way of data storage (patient number not available), the data of campus Leuven didn't allow to identify the age and sex of the patient nor the proportion of patients with repeated falls. The analyses regarding age, sex and repeated falls were only performed on the subsample of campus Kortenberg.

## Results

### Prevalence of registered falls

An overview of the data regarding prevalence is shown in Table 1.

**Table 1:** Data on the prevalence of registered falls.

	**Jul–Dec 2013**	**2014**	**2015**	**2016**	**2017**	**2018**	**Jan–Jun 2019**	**Total**
Falls	410	705	635	665	774	733	403	4,324
Admissions	2,043	3,928	4,092	3,961	3,841	3,903	1,862	23,630
Patient days	82,452	164,261	169,638	166,118	162,525	165,537	80,373	990,904
Falls/admission	0.2	0.2	0.2	0.2	0.2	0.2	0.2	0.2
Falls/1.000 patient days	4.9	4.3	3.7	4.0	4.8	4.4	5.0	4.4

A total of 4,324 falls (3,251 for campus Kortenberg and 1,073 for campus Leuven), 23,630 admissions and 990,904 patient days were registered during the study period. This makes a total of 0.2 falls/admission and 4.4 falls/1,000 patient days.

### Prevalence of falls resulting in injury

The majority of falls had no (64%) or mild (24%) physical consequences. Five percent had moderate consequences and required investigation. Only 0.5% of the incidents were considered as severe. None of the incidents were fatal. For 5.7% of the incidents, the severity rate was not reported in the PiMS form. Supplementary Table 1 shows an overview of the data regarding the prevalence of falls resulting in injury.

### Factors associated with falls

#### Cause

An overview of the frequencies of possible causes is shown in Table 2. The health status of the patient (51.9%) and the behavior of the patient (45.0%) were reported as the two most common causes of the fall. In 6.7% of registered falls the fall was considered medication-related. Note that multiple options could be indicated on the report form.

**Table 2:** Data on the frequencies of possible causes.

	**Number of falls**	**Number of falls within this group/total number of falls (*n* = 4.324)**
Health status	2,248	51.9%
Behavior	1,946	45.0%
Environment	553	12.8%
Medication	288	6.7%
Intoxication	17	0.4%
Other factors	396	9.2%
Unknown	38	0.9%
No data	792	18.3%

Multiple options could be indicated per incident.

#### Location of fall

More than half of the patients (51.5%) fell in their own room. The other half fell mainly in the shared living area of the hospital ward (18.6%), in the corridor of the ward (15.7%) or somewhere else in the ward (5.7%). A small percentage of patients (5.4%) fell in the bathroom or on the toilet. The other patients (3.1%) fell somewhere outside of the ward.

#### Sex

**Figure 1:**
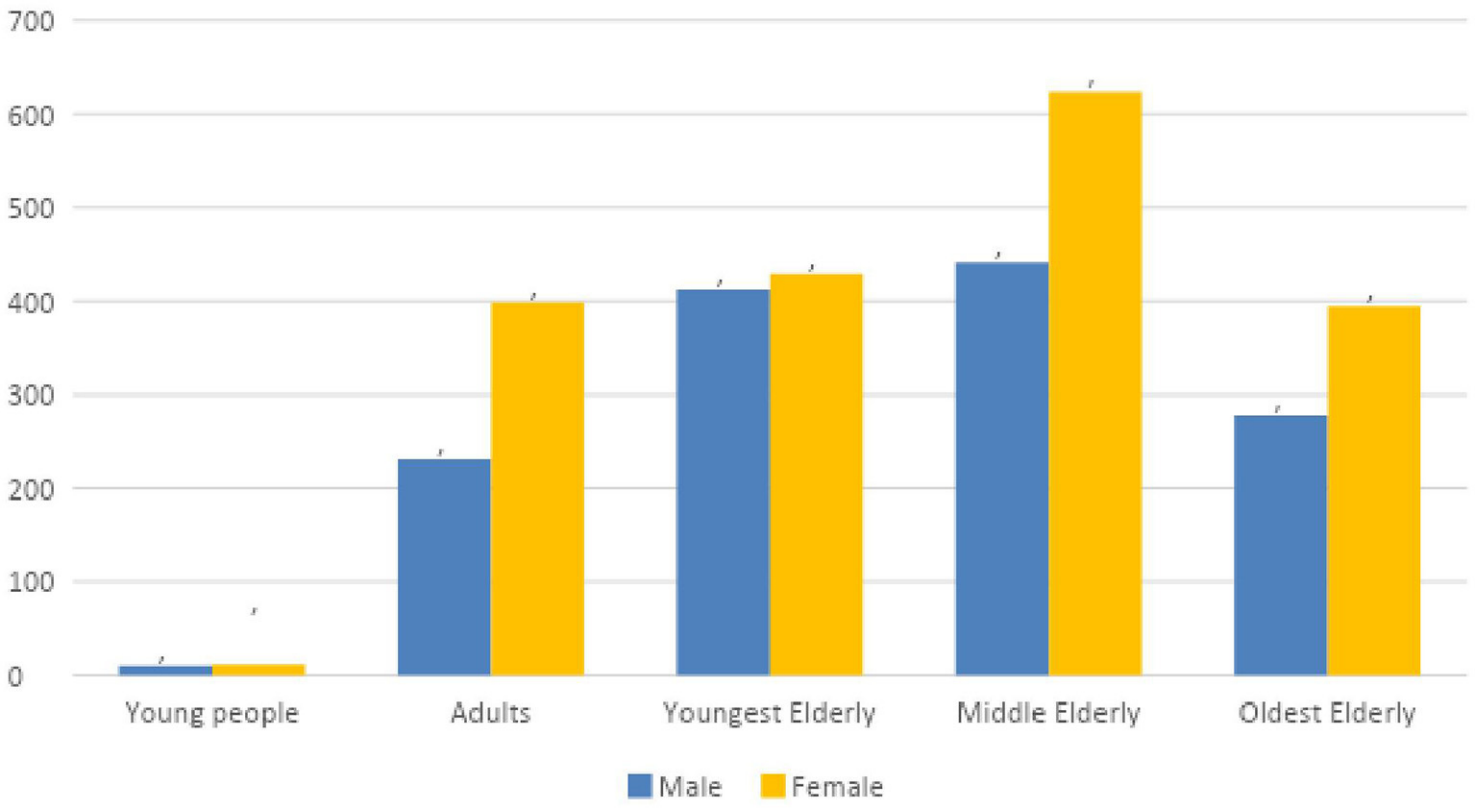
Distribution of age and sex of reported falls (campus Kortenberg). Representation of the distribution of gender and age. Incidents where age and/or gender were unknown were excluded.

In 57.5% of registered falls (*n* = 1,859) the patient was female. Figure 1 shows that the man/women ratio was more or less consistent when looking at gender in relation to age. For example, in the group of the *middle elderly* and the *oldest elderly* respectively 58.6 and 58.7% of these patients were female. For the group of young people this was only 54.4%, in the adult group 63.3%.

#### Age

Eighty-six percent of falls took place in a hospital ward for elder patients, 13% in a ward for adults and 1% in the children's department. Data regarding age are shown in Table 3. Mean age of the patients with a registered fall was 72.2 years (SD 15, 5). The youngest patient was 13 years old, the oldest 100 years. Eighty percent of the falls involved a patient over the age of 65. Within this group, most incidents occurred in the *middle elderly* (32.9%), followed by the *youngest elderly* (26.0%) and the *oldest elderly* (20.9%). Only 25.0% of elderly patients suffered physical consequences because of a fall, as opposed to 31.4% of adults and 68.2% of young people. In the group of elderly patients, most physical injuries were observed in the group of oldest elderly. Nearly 10% of all patients admitted during the study period fell at least one time during their stay and with increasing age proportionally more falls were registered.

**Table 3:** Data on the number of falls, number of falls resulting in physical injury, number of patients with a fall and number of admissions, expressed in relation to age.

	**Number of falls**	**Number of falls/total number of falls**	**Number of falls resulting in physical injury**	**Number of falls resulting in physical injury/number of falls within this group**	**Number of patients with at least one fall**	**Number of unique patients with at least one admission**	**Number of patients with at least one fall/number of unique patients**
Young people (<18)	22	0.7%	15	68.2%	19	677	2.8%
Adults (18–64)	632	19.5%	196	31.0%	269	7,091	3.8%
Youngest elderly (65–74)	841	26.0%	176	20.9%	171	919	18.6%
Middle elderly (75–84)	1,066	32.9%	277	26.0%	322	1,231	26.2%
Oldest elderly (>85)	679	20.9%	194	28.6%	251	834	30.1%
Total	3,240^*^	100%	858	26.5%	1,032	10,570	9.8%

* A total of 3,251 falls was registered on this campus during the study period, however in 11 incidents the age of the patient was not available (unknown reason).

This table only includes data of the subsample of campus Kortenberg.

### Prevalence of repeated falls and associated factors

Almost half of the patients (47.3%) were identified as repeated fallers.

Thirty-six percent of the repeated fallers fell twice during the 12 months study period. Twenty percent fell three times and 13.3% four times (with a maximum of 12 months between two consecutive falls). Sixteen patients (3.3%) fell twenty times or more during their stay.

The probability of recurrence was estimated by the health care professional who reported the incident: 41.3% was reported as almost certain to be repeated, 34.2% as probably and only 1.1% as unlikely.

Mean age of the patients with a repeated fall was 73.5 years (SD 13, 7). The youngest patients was 16 years old, the oldest 100 years. Table 4 shows that repeated falls mainly occurred in people over the age of 65. When the number of repeated falls was compared with the total number of falls on this campus, it was found that the youngest elderly fell most frequently.

**Table 4:** Data on the age distribution in the ‘repeated falls’ group (campus Kortenberg).

	**Number of falls**	**Number of repeated falls**	**Number of patients with a repeated fall**	**Number of repeated falls/number of falls**	**Number of falls/total falls**
Young people (<18)	22	5	2	22.7%	0.7%
Adults (18–64)	632	434	78	68.7%	19.5%
Youngest elderly (65–74)	841	765	102	91.0%	26.0%
Middle elderly (75–84)	1,066	914	179	85.7%	32.9%
Oldest elderly (>85)	679	552	128	81.3%	21.0%
Total	3,240^*^	2,670	489	82.4%	100%

* A total of 3,251 falls was registered on this campus during the study period, however in 11 incidents the age of the patient was not available (unknown reason).

This table only includes data of the subsample of campus Kortenberg.

Low numbers of serious incidents were observed in this repeated falls group, compared to the full dataset of this campus (see Table 5).

**Table 5:** Data on the physical consequences in the ‘repeated falls’ group (campus Kortenberg).

	**Number of falls**	**Number of repeated falls**	**Number of repeated falls/number of falls**	**Number of falls/total falls**
None	2,274	1,966	86.5%	69.9%
Mild	707	522	73.8%	21.8%
Moderate	147	111	75.5%	4.5%
Severe	15	3	20.0%	0.5%
Not reported	108	68	63.0%	3.3%
Total	3,251	2,670	82.1%	100%

This table only includes data of the subsample of campus Kortenberg.

## Discussion

This study showed a fall ratio of 4.4 falls per 1,000 patient days. This result is in line with some previous studies (43, 44) showing ratios ranging from 3.7 to 4.6. In contrast, Turner et al. (45) found a higher ratio of 8.6. Rao et al., in their systematic review on the incidence of falls in psychiatric inpatients in China, observed a significantly lower incidence. However, underreporting could not be excluded because in some Chinese hospitals falls were considered as minor accidents (37).

No incident was fatal and only 0.5% of falls resulted in severe injury. Age and gender of these falls were often unknown (respectively 19 and 17 of the 23 falls), making it impossible to define risk groups.

In contrast to the results of Tay et al. (38) and Poster et al. (44), where respectively 14 and 50% of the incidents had no physical consequences, in this study 65% of the falls were registered without any physical injury. Whether this could be a result of the fall prevention policy in our hospital, cannot be answered.

Evidence suggests that patients often fall on their way to or in the bathroom (33, 44, 45). In this study, more than half of the registered falls took place in the patient's room or in the bathroom/on the toilet (respectively 52 and 5%). A similar observation was made by Poster et al., finding percentages of respectively 42 and 10%. This also applies to patients in non-psychiatric hospitals (19, 44).

Looking at the number of patients with a fall and the number of admissions in relation to age, proportionally more falls were registered with increasing age. This shouldn't be surprising as several studies have already pointed out that older age is an important risk factor for falling (43, 44, 46).

Nearly 50% of patients (47%) fell at least twice within the next 12 months. A history of falling is an important risk factor for falls, especially for those resulting in physical injury (18, 19, 47). In order to minimize the chance of recurrence, it is important to map out the situation and circumstances of previous falls as accurately as possible (48).

### Strengths and limitations

The relatively large sample size was a strength of this study. A total of 4,324 falls was studied, without any exclusion for age or diagnosis. Moreover, the elderly group was divided by age in different subgroups (youngest, middle and oldest elderly), providing additional insights, namely that the middle elderly fell more, but the oldest elderly suffered more physical consequences.

A number of limitations must also be taken into account when interpreting the results. A first limitation is the lack of a control group. A second limitation is the fact that the study was only performed in one hospital of this specific region. The third limitation are the characteristics of and differences between the report forms, which were not primarily designed for use in a study context. Due to the fact that this study made use of voluntary reporting forms, underreporting of the number of incidents cannot be excluded (38). In addition, non-mandatory fields were often not filled in, resulting in the fact that the analysis of certain elements was limited to files with incomplete data. The last limitation is that the severity level of physical consequences was not always known at the time of the registration of the fall, which could lead to an underestimation of the severity of injuries.

Further research should include patient diagnosis and length of hospital stay, as depression has been identified as an independent risk factor (apart from the intake of psychotropic medication) (19, 38, 49, 50) and the first week of admission has been shown to be a specific high-risk period that requires extra vigilance (43). On the other hand, with increasing length of hospital stay, other risk factors that were initially not relevant can arise and caregivers can become less attentive in comparison to the start of the admission (44, 51).

Although this study was purely descriptive, our data provide a good basis to measure post-intervention changes in the future, as it was recently shown that mainly patient and staff education and personalized falls prevention strategies can reduce hospital falls (52, 53), it would be interesting to see if such interventions would reduce fall incidents in our hospital.

## Conclusion

In summary, our results generally were in line with the ratios found in the existing literature on fall prevalence in psychiatric hospitals. Certain links with age were observed. Nearly 80% of falls involved a patient over the age of 65. On the other hand, young people showed more physical consequences from a fall compared to adults and elderly, suggesting that all age categories deserve sufficient attention in fall prevention policies.

## Data availability statement

The raw data supporting the conclusions of this article will be made available by the authors, without undue reservation.

## Ethics statement

The studies involving human participants were reviewed and approved by Ethical Research Committee UZ Leuven - KU Leuven. Written informed consent from the participants' legal guardian/next of kin was not required to participate in this study in accordance with the national legislation and the institutional requirements.

## Author contributions

All authors listed have made a substantial, direct, and intellectual contribution to the work and approved it for publication.

## Conflict of interest

The authors declare that the research was conducted in the absence of any commercial or financial relationships that could be construed as a potential conflict of interest.

## Publisher's note

All claims expressed in this article are solely those of the authors and do not necessarily represent those of their affiliated organizations, or those of the publisher, the editors and the reviewers. Any product that may be evaluated in this article, or claim that may be made by its manufacturer, is not guaranteed or endorsed by the publisher.
