# Supplementary material for: Prevalence and characteristics of registered falls in a Belgian University Psychiatric Hospital
Source: Front Public Health. 2022 Oct 28;10:1020975. doi: 10.3389/fpubh.2022.1020975 (PMC9651969; doi:10.3389/fpubh.2022.1020975)
Supplement: Supplementary file 1 [file Image_1.pdf]

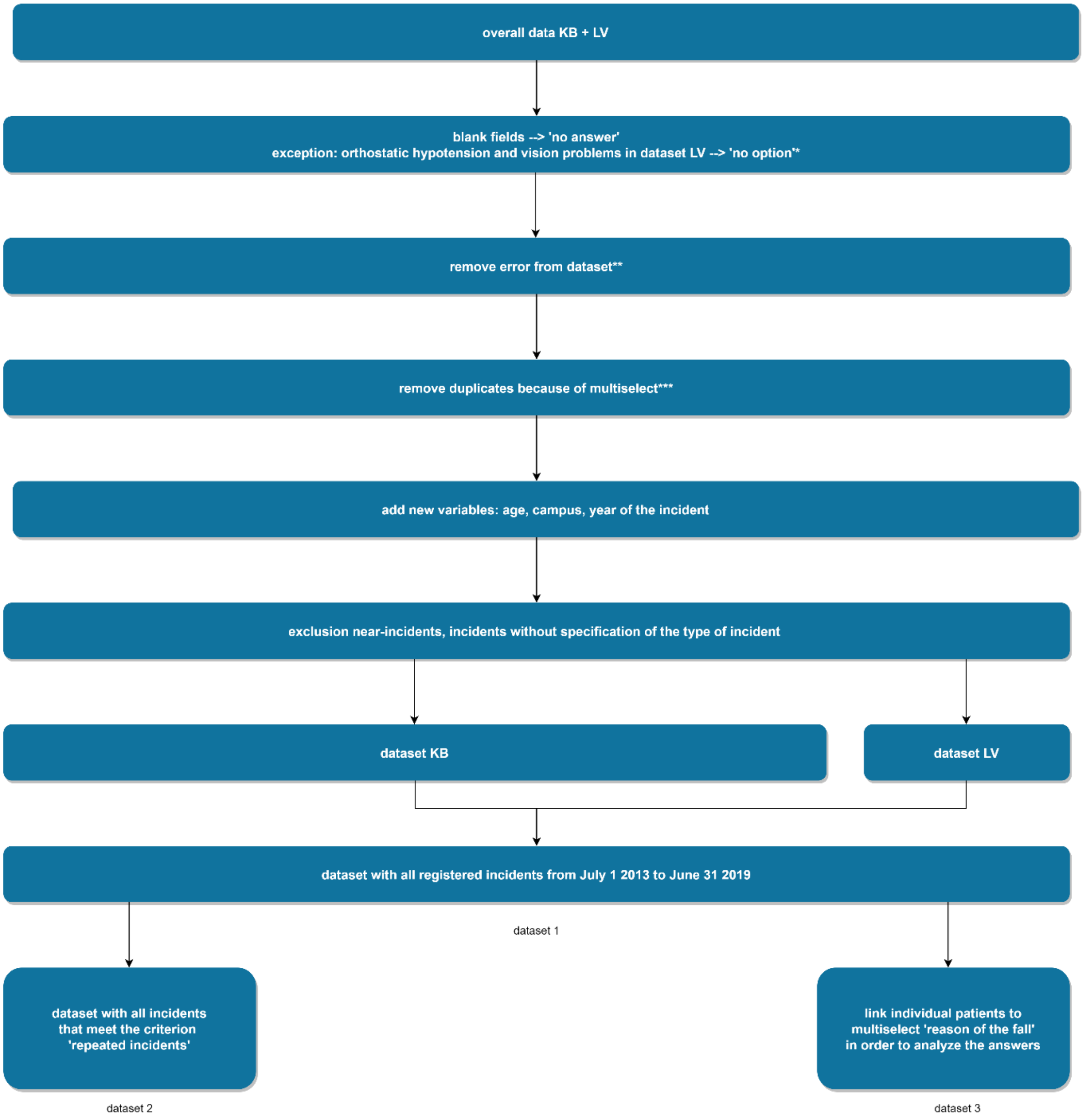

* Whether the patient suffered from orthostatic hypotension and/or visual disturbances at the time of the fall was not specifically asked on the report form of campus LV, which is why this variable was filled in for this campus with 'no option' instead of 'no answer'.

** Due to an error, two incidents occurred multiple times (2.190) in the 2016-2019 datasets. How this error came about is not known.

*** Multiselects are questions where the health care provider could indicate multiple answers. Each answer was displayed in a new row, where the other answers were copied identically. To simplify the analysis, each answer was reduced to 1 row, as everything except the multiselect was identical.

Supplementary Figure 1. More detailed overview of data processing.
