# Supplementary material for: Prevalence and characteristics of registered falls in a Belgian University Psychiatric Hospital
Source: Front Public Health. 2022 Oct 28;10:1020975. doi: 10.3389/fpubh.2022.1020975 (PMC9651969; doi:10.3389/fpubh.2022.1020975)
Supplement: Supplementary file 2 [file Table_1.docx]

|  | Number of falls | Number of falls within this group / Total number of falls (n=4.324) |
| --- | --- | --- |
| None | **2,790** | **64.5 %** |
| Mild | **1,048** | **24.2 %** |
| Moderate | **217** | **5 %** |
| Severe | **23** | **0.5 %** |
| Not Reported | **246** | **5.7 %** |

**Supplementary Table 1.** Data on the frequencies of falls resulting in injury
